# Primary melanoma of the bladder: case report and review of the literature

**DOI:** 10.1186/s12957-022-02753-5

**Published:** 2022-09-07

**Authors:** Jin-Dong Dai, Ben He, Zhi-Hong Liu, Ming Shi, Peng-Fei Shen

**Affiliations:** 1 Department of Urology, Institute of Urology, West China Hospital, Sichuan University, Chengdu, 610041 China; 2 Department of Urology, The Third People’s Hospital of Chengdu, The Affiliated Hospital of Southwest Jiaotong University, Chengdu, Sichuan People’s Republic of China

**Keywords:** Melanoma, Bladder tumor, Treatment, Case report

## Abstract

**Background:**

Primary melanoma of the bladder is extremely rare and has been sporadically reported in case reports. Its incidence, diagnosis, treatment, and outcomes are still unclear.

**Case presentation:**

We report a 67-year-old female patient who presented with hematuria and was diagnosed with primary melanoma of the bladder by transurethral resection. No distant metastasis was detected by fluorodeoxyglucose positron emission tomography-computed tomography (PET-CT). After a multidisciplinary discussion, the patient received laparoscopic radical resection of the bladder tumor. There was no tumor recurrence or distant metastasis after 15 months of follow-up.

**Conclusion:**

Primary melanoma of the bladder is easily confused with urothelium carcinoma in morphology. The immunohistochemical is crucial in diagnosis. Because of a lack of in-depth understanding of primary melanoma of the bladder, the “gold standard” treatment has not been set. We would like to provide some rare information about it and discuss the proper treatment strategy for this rare disease.

**Supplementary Information:**

The online version contains supplementary material available at 10.1186/s12957-022-02753-5.

## Background

Primary melanoma of the bladder is extremely rare and accounts for less than 0.2% of all reported melanoma cases. It has been reported that 96–95% of all primary melanomas arise from the skin and have a lethal effect on patients. Thus far, up to 30 corresponding cases have been reported [1]. However, no standard treatment has been established for local or advanced primary bladder melanoma.

Transurethral resection (TUR), partial cystectomy (PC), and radical cystectomy (RC) are ordinarily regarded as optional treatments for primary melanoma of the bladder. Although the tumor size and depth of invasion might determine the prognosis in primary disease, the overall survival time is less than 3 years. Because of the risk of recurrence in the bladder or systemic metastasis after local treatment in patients with primary bladder melanoma, RC is recommended in some cases [2]. However, some studies reported that RC did not achieve the expected positive effect on survival [3]. Thus, we report the positive effect of RC on survival in a patient with confirmed primary bladder malignant melanoma.

### Case presentation

**Fig. 1:**
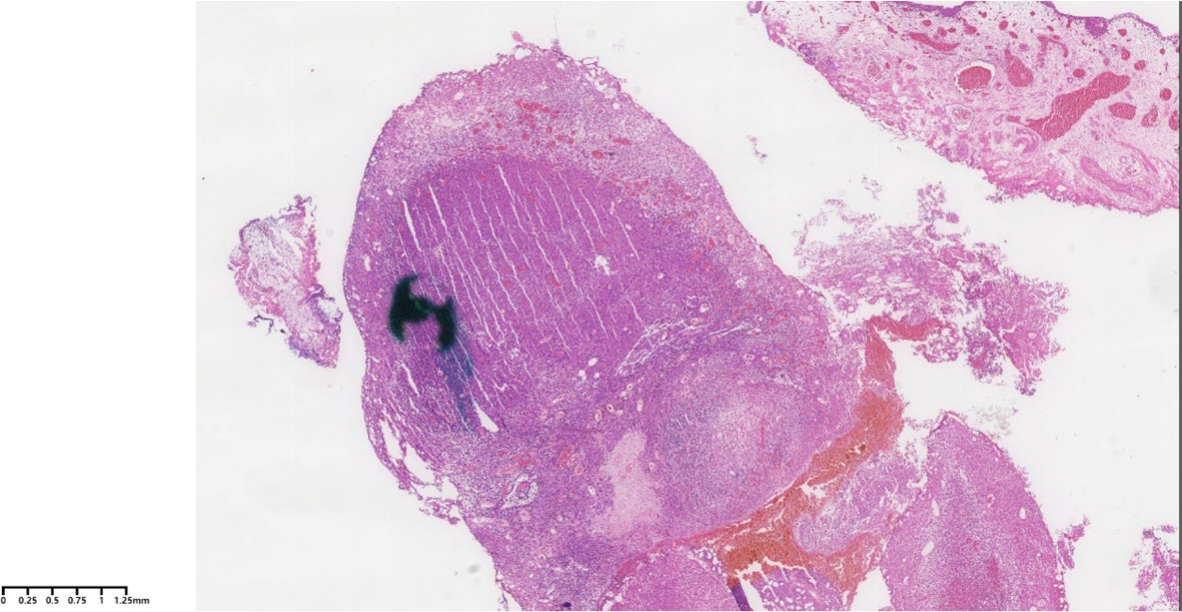
Pathological evaluation of the first operation (TUR) by H&E (hematoxylin-eosin staining); HES × 10

A 67-year-old female patient with a complaint of gross hematuria underwent a cystoscopy, and a mass lesion approximately 1 cm in size was detected on the left side of the bladder neck. A detailed outpatient consultation revealed that the non-smoker patient had no additional systematic diseases and was not taking any medications. A subtotal hysterectomy has been performed more than 10 years ago due to the myoma of the uterus. In addition, the physical examination did not show further positive findings. After all preoperational preparations were ready, a complete TUR of the bladder tumor was performed in the primary hospital. Based on the pathological evaluation by HE (hematoxylin-eosin) staining, a high-grade urothelial carcinoma was initially diagnosed (Fig. 1).

**Fig. 2:**
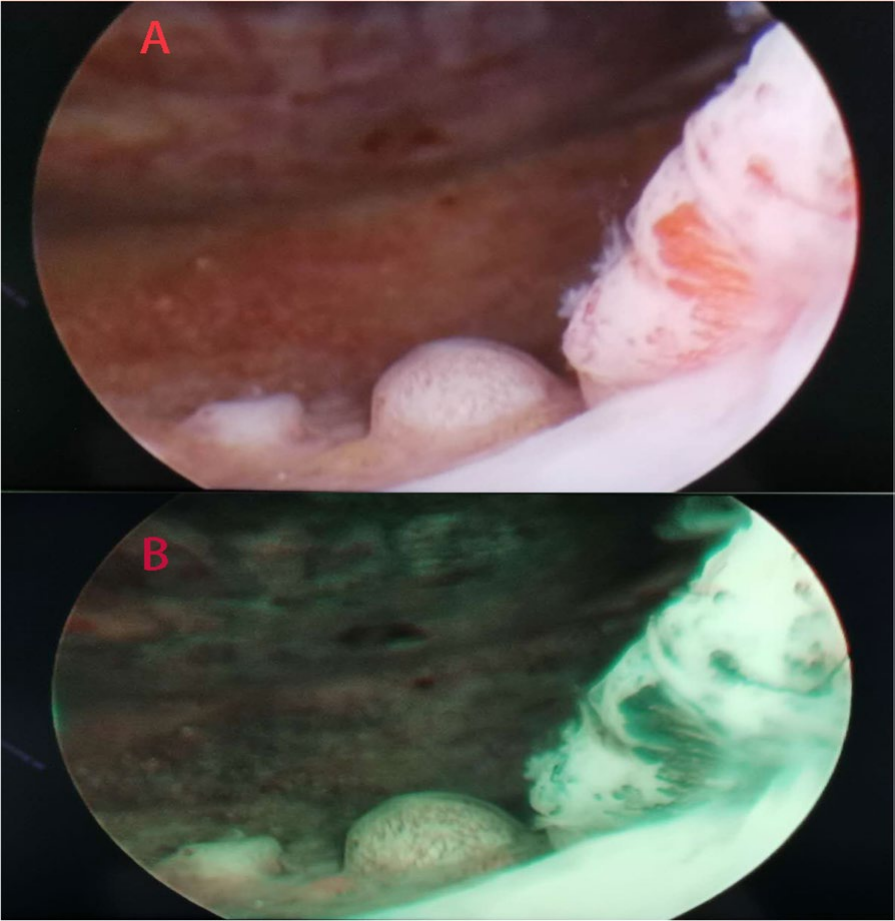
A Tumor lesion in the bladder seen endoscopically (TUR). **B** Tumor lesion in the bladder seen endoscopically under narrow-band imaging (NBI). The length of the biggest lesion was about 1.5cm

**Fig. 3:**
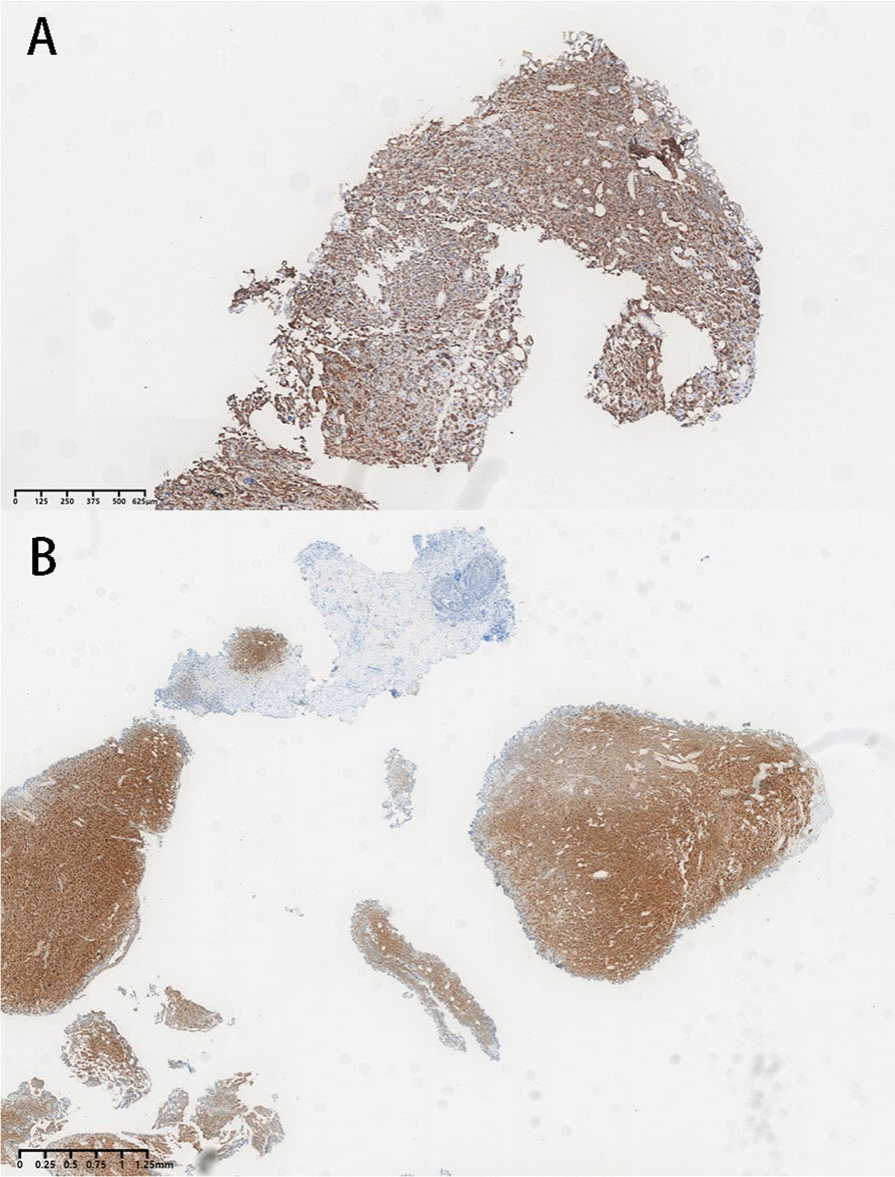
The gross specimen of the second operation (TUR). **A** Positive staining with HMB 45; HES X15. **B** Positive staining with S 100; HES × 10

To prevent the recurrence of the bladder tumor, a bladder perfusion with gemcitabine was then performed. The hematuria reappeared after 4 circles of perfusion of the bladder. After routine laboratory tests, the following parameters were obtained: hemoglobin 13.7 g/dL; creatinine 49 umol/L; urine analysis, leukocytes 30/HPF; and erythrocytes 9/HPF. After all the needed preoperational preparations were completed, a TUR of the bladder tumor was performed again in our hospital (Fig. 2). After pathological evaluation by the HE staining method mentioned above, the tumor was then examined by immunohistochemical (IHC) staining. Mart-1, HMB-45, and S-100 were positive, while cytokeratin was negative (Fig. 3). The final pathology was reported as primary bladder malignant melanoma.

**Fig. 4:**
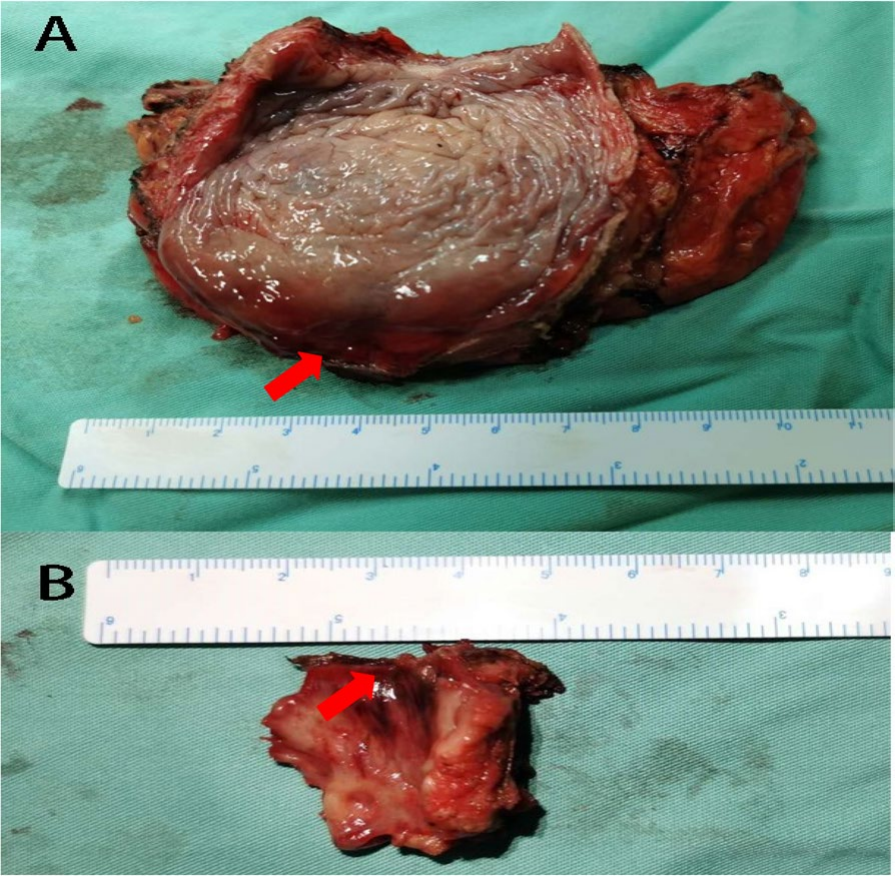
Pathological evaluation of the third operation (radical cystectomy). **A** The operative specimen of the bladder. The red arrow marks the point of the neck of the bladder. **B** The operative specimen of the urethra. The red arrow marks the point of the neck of the bladder

One month after TUR, fluorodeoxyglucose (FDG) positron emission tomography-computed tomography (PET-CT) revealed no distant metastasis in the body of the patient (Supplemental figure 1). To make a treatment plan and prognosis assessment, the patient was discussed by members of the institutional multidisciplinary uro-oncologic disease management team, who proposed a treatment plan. By fully communicating with the patient and her family members regarding the patient’s condition and treatment plan, a RC with double cutaneous ureterostomy was performed. Fortunately, no tumor in the bladder was found after pathological evaluation (Fig. 4). Follow-up of the patient lasted 15 months, and no recurrence or metastasis was found.

## Discussion and conclusion

Primary bladder malignant melanoma is extremely rare in the genitourinary tract, especially in the bladder. Since Wheelock and colleagues reported the first case of primary bladder malignant melanoma of the bladder in 1942, approximately 31 cases of primary bladder melanoma have been reported in the authoritative medical literature. All the reported cases are listed in Table 1. The median age of patients was 61 (from 7 to 87) years old, and 17/31 patients were male. In addition, the follow-up time varied from 0.5 to 144 months (a median of 14 months), during which only 13/31 (41.9%) patients were still alive. It has been reported that bladder metastasis of malignant melanoma is more common than primary bladder malignant melanoma. Furthermore, renal and bladder metastases have been reported in 45% and 18% of patients dying of melanoma, respectively [2].

**Table 1:** Primary melanomas of the bladder reported in literature

Reference	Year	Age (year)	Gender	Treatment	Follow-up (Months)	Outcomes
Wheelock [4]	1942	67	Female	Partial cystectomy	36	Died
Su and Prince [5]	1962	61	Female	None	2	Died
Ainsworth et al. [6]	1976	65	Female	Radical cystectomy	17	Alive
Willis et al. [7]	1980	57	Female	Radical cystectomy	36	Died
Anichkov and Nikonov [8]	1982	48	Male	Partial cystectomy	12	Died
Anichkov and Nikonov [8]	1982	46	Male	Radical cystectomy	3	Alive
Ironside et al. [ 9]	1985	56	Male	None	8	Died
Goldschmidt et al. [ 10]	1988	53	Female	Partial cystectomy	7	Died
Goldschmidt et al. [ 10]	1988	56	Female	None	6	Alive
Philippe et al. [ 11]	1989	77	Male	TUR	-	-
Van Ahlen et al. [ 12]	1992	81	Male	Radical cystectomy, radiotherapy, interferon-alpha	24	Died
Lund et al. [ 13]	1992	81	Female	Local excision, radiotherapy, chemotherapy	15	Alive
Kojima et al. [ 14]	1992	63	Female	TUR + Chemotherapy	18	Died
Lange-Welker et al. [ 15]	1993	75	Male	Partial cystectomy	3	Died
Mourad et al. [ 16]	1993	34	Male	Radical cystectomy	12	Alive
Niederberger and Lome [17]	1993	53	Male	Radical cystectomy	18	Alive
De Torres et al. [ 18]	1995	44	Male	Radical cystectomy	14	Died
Tainio et al. [ 19]	1999	52	Male	TUR	8	Died
Garcia Montes et al. [ 20]	2000	44	Female	TUR	144	Alive
Khalbuss et al. [ 21]	2001	82	Female	Radiotherapy	16	Died
T. Hsu and Y. Hsu [22]	2002	73	Male	TUR+ intravesical BCG and ReTUR at 2-7-9 months	16	Alive
Baudet et al. [ 23]	2005	7	Female	Partial cystectomy	84	Alive
Pacella et al. [ 24]	2006	82	Male	TUR	9	Died
Sundersingh et al. [ 25]	2011	56	Male	Radical cystectomy and pelvic excision four months later	10	Alive
El Ammari et al. [ 26]	2011	71	Male	TUR	5	Died
Truong et al. [ 27]	2013	84	Female	TUR + Ipilimumab	-	-
Otto et al. [ 28]	2017	52	Male	TUR + Interferon/dacarbazine	18	Died
Barillaro et al. [ 29]	2018	72	Male	Radical cystectomy + Nivolumab	16	Alive
Bumbu GA, et al. [30]	2019	80	Male	TUR + Chemotherapy	6	Died
Monica K, et al. [ 31]	2019	87	Female	None	0.5	Died
Mercimek MN, et al. [2]	2019	39	Female	Partial cystectomy + BPLND	52	Alive

*TUR*, transurethral resection; *BCG*, Bacillus of Calmette Guerin; *BPLND*, bilateral pelvic lymph node dissection

The symptoms caused by this tumor vary and depend on its location [32]. The patient in our report complained of hematuria for 1 month. Gross hematuria is one of the most frequent presenting symptoms, depending on the size and location of the tumor in the bladder. It was reported that recurrent urinary tract infections might be an initial symptom in some patients with primary bladder melanoma [32].

In ordinary clinical practice, the diagnosis of primary melanoma of the bladder is complicated. In this case, the tumor was originally misdiagnosed as a high-grade urothelial carcinoma. IHC staining is crucial to make an accurate diagnosis of this disease, and immunohistochemical staining such as HMB-45, Melan-A, and S-100 are helpful in diagnosis during the pathological examination. Thus far, some diagnostic criteria for primary melanoma in the bladder have been established as follows [30]: (1) absence of any previous skin lesions, (2) cutaneous malignant melanoma, (3) primary visceral malignant melanoma, (4) recurrence pattern showing consistency with the primary tumor diagnosis, and (5) atypical melanocytes at the tumor margin upon microscopic examination.

It is generally known that no standard treatment has been established for primary melanoma in the bladder. The PC, RC, and TUR were potential surgical plans. TUR was always considered a conservative treatment for some patients. The tumor is apt to recurrence, and metastasis after TUR, therefore, refers to the treatment of urothelium carcinoma; BCG instillation was used to decrease the recurrence of carcinoma after TUR. There was only one patient treated with Bacillus of Calmette Guerin (BCG) after TUR; however, its effect after TUR still requires further explore [22]. Some case studies revealed that chemotherapy could be considered a therapy for patients after surgery. For the two patients without distant metastasis, they were treated with chemotherapy after TUR, and the patients died after 18 and 6 months, respectively [ 14, 30]. RC was carried out on 10 patients, and the survival rate was 60% (6/10) at a median follow-up of 15.5 months.

Two patients with suspicious distant metastasis received immune checkpoint inhibitors (ICIs) after surgery, and one of the patients who received Nivolumab after RC was alive at 16 months [29]. Nivolumab plus Ipilimumab or Nivolumab alone, blocking the interaction between the programmed cell death PD1, and its ligand PD-L1 have been reported to be effective in antitumor response in melanoma [31, 33, 34]. ICIs might be considered for the primay bladder melanoma patients with distant metastasis after surgery.

In this case report, the patient was informed regarding all corresponding treatment options in advance and was managed in a pre-emptive manner by carrying out RC following transurethral resection of the bladder tumor.

In conclusion, primary melanoma of the bladder is easily confused with urothelium carcinoma in morphology. The IHC is crucial in diagnosis. Because of the lack of adequate evidence about the treatment of patients with primary melanoma of the bladder, no “Gold standard” treatment has been set. We would like to provide some rare information about primary bladder melanoma and discuss the appropriate treatment strategy for this rare disease.

## Supplementary Information

**Additional file 1: Supplemental Figure S1**: The patients’ preoperative PET/MRI scan. The red arrow marks the point of the tumor in neck of bladder.

**Additional file 2: Supplemental Figure S2**: The pictures of patient’s hematuria sample.

## Data Availability

Raw data for the figures are not publicly available to preserve individuals’ privacy under the European General Data Protection Regulation.

## Abbreviations

BCG: Bacillus of Calmette Guerin
FDG: Fluorodeoxyglucose
PET-CT: Positron emission tomography-computed tomography
H&E staining: Hematoxylin-eosin staining
IHC: Immunohistochemical
PC: Partial cystectomy
RC: Radical cystectomy
TUR: Transurethral resection
ICIs: Immune checkpoint inhibitors
