# Supplementary figures and images for: Primary melanoma of the bladder: case report and review of the literature
Source: World J Surg Oncol. 2022 Sep 7;20:287. doi: 10.1186/s12957-022-02753-5 (PMC9454232; doi:10.1186/s12957-022-02753-5)

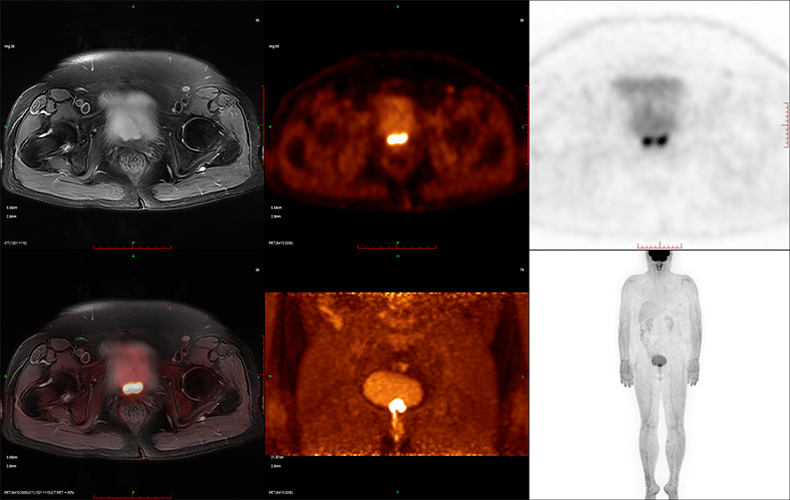

Supplement: Supplementary file 1 — Additional file 1: Supplemental Figure S1: The patients’ preoperative PET/MRI scan. The red arrow marks the point of the tumor in neck of bladder. [file 12957_2022_2753_MOESM1_ESM.jpg]

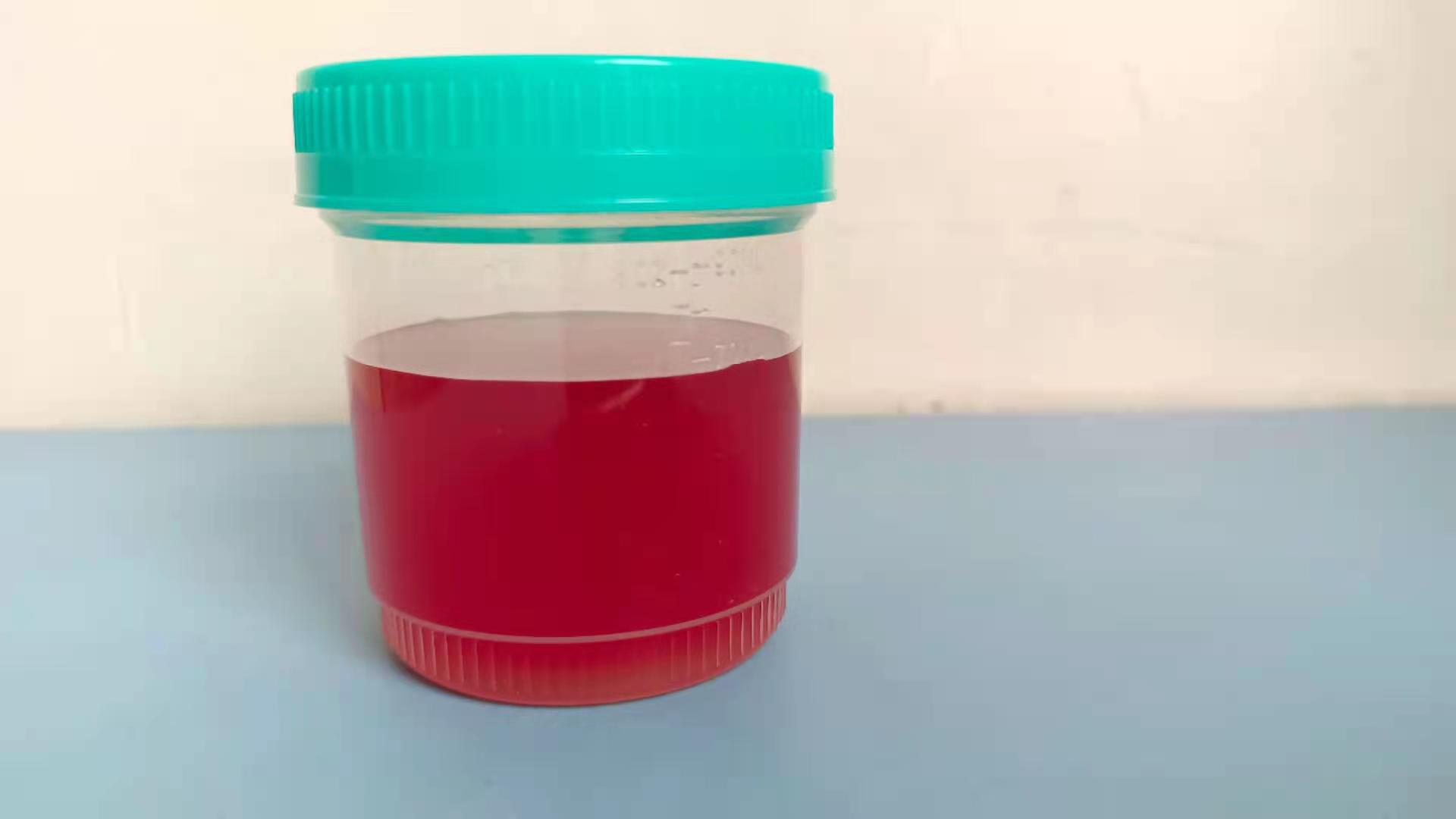

Supplement: Supplementary file 2 — Additional file 2: Supplemental Figure S2: The pictures of patient’s hematuria sample. [file 12957_2022_2753_MOESM2_ESM.jpg]
